# Two Polymorphisms in the Fractalkine Receptor CX3CR1 Gene Influence the Development of Atherosclerosis: A Meta-Analysis

**DOI:** 10.1155/2014/913678

**Published:** 2014-08-26

**Authors:** Jian Wu, Rui-Xing Yin, Quan-Zhen Lin, Tao Guo, Guang-Yuan Shi, Jia-Qi Sun, Shao-Wen Shen, Qing Li

**Affiliations:** ^1^Department of Cardiology, Institute of Cardiovascular Diseases, The First Affiliated Hospital, Guangxi Medical University, 22 Shuangyong Road, Nanning, Guangxi 530021, China; ^2^Department of Internal Medicine, Affiliated Shanghai First People's Hospital, Shanghai Jiao Tong University, Shanghai 200080, China

## Abstract

*Background*. The associations between the Fractalkine receptor (CX3CR1) gene T280M (rs3732378) and V249I (rs3732379) polymorphisms and atherosclerosis (AS) risk are conflicting. The aim of this meta-analysis was undertaken to assess their associations. *Methods*. PubMed, Embase, Web of Science, Medline, Cochrane database, and CNKI were searched to get the genetic association studies. All statistical analyses were done with Stata 11.0. *Results*. Twenty-five articles involving 49 studies were included in the final meta-analysis. The analysis showed that the 280M allele carriers of the *CX3CR1* T280M polymorphism decreased the risk of AS and coronary artery disease (CAD) in the heterozygous state but increased the risk of ischemic cerebrovascular disease (ICVD) in the homozygote state. The 249I allele carriers of the *CX3CR1* V249I polymorphism decreased the risk of AS and CAD in the heterozygous state. The V249I-T280M combined genotype VITM and IITM also decreased the risk of AS. *Conclusions*. The present meta-analysis suggests that the *CX3CR1* T280M and V249I polymorphisms are associated with the susceptibility to AS. However, the results should be interpreted with caution because of the high heterogeneity in the meta-analysis.

## 1. Introduction

Atherosclerosis (AS) is a multifactorial, progressive disease and a major global cause of morbidity and mortality. Atherosclerosis-related cardiovascular diseases, such as coronary artery disease (CAD), acute myocardial infarction (AMI), and ischemic cerebrovascular disease (ICVD), are the causes of death in almost 50% of cases in developed countries [1]. The exact mechanisms of AS are indistinct. Many studies indicate that the genetic factors play a significant role in the development of AS [2–5]. Until recently, much attention has been focused on this field, and the association of the Fractalkine (FKN) receptor (CX3CR1) gene polymorphisms and AS has been extensively studied [6–31].

It has recently been accepted that AS is predominantly an inflammatory process [32, 33] and begins with a fatty streak, which is made up almost entirely of monocyte-derived macrophages [33, 34]. During the process of vascular inflammation, chemokines and adhesive molecules exert a vital role via mediating the activation of inflammatory cells and their aggregation or adhesion to vascular walls [35, 36]. FKN is a special chemotactic factor existing in both membrane-bound and soluble forms [37]; the expression of FKN and its receptor CX3CR1 is upregulated in AS lesions [38–40], and the severity of AS is greatly improved by inhibiting their expression [40–42], suggesting that the FKN/CX3CR1 is closely correlated to AS. Given the crucial role of* CX3CR1* in the inflammatory process, the mutations in the* CX3CR1* may also play a significant role in the development of atherosclerotic diseases. Recently, a number of molecular epidemiological studies have been done to evaluate the associations between the* CX3CR1* gene polymorphisms (T280M and V249I) and the risk of atherosclerotic diseases [6–31]. However, the results of different studies are inconsistent, possibly due to small sample sizes in the individual studies. In 2009, Apostolakis et al. [43] performed a meta-analysis to evaluate the association between the* CX3CR1* T280M and V249I polymorphisms and CAD and demonstrated that the* CX3CR1* 280M allele was associated with a reduced risk of CAD in the heterozygous state and 249I-280M haplotype had a atheroprotective effect on CAD. However, they just studied the 280M allele and 249I-280M haplotype of* CX3CR1* in Caucasians. Considering the meta-analysis only focused on the association of the* CX3CR1* polymorphism with the single atherosclerotic disease, we, therefore, performed this meta-analysis of all the studies available now to get a more precise estimation of the associations between the* CX3CR1 *T280 M (rs3732378) and V249I (rs3732379) polymorphisms and overall AS risk.

## 2. Materials and Methods

### 2.1. Literature Search and Selection

This meta-analysis followed the Preferred Reporting Items for Systematic Reviews and Meta-analyses (PRISMA) criteria [45]. Eligible literatures published before the end of March 1, 2014, were identified by the search of PubMed, Embase, Web of Science, Medline, Cochrane database, and CNKI. Library uses combinations of the following keywords: “chemokine receptor 1” or “FKN” or “CX3CR1” or “fractalkine” and “polymorphism” or “mutation” or “variant” or “variation” or “genotype” and “coronary artery disease” or “CAD” or “coronary heart disease” or “CHD” or “myocardial infarction” or “MI” or “ischemic cardiovascular disease” or “ischemic cardiovascular events” or “ischemic stroke” or “IS” or “cerebrovascular disease” or “ischemic cerebrovascular events” or “cerebral infarction” or “cerebral ischemia” or “brain infarction” or “carotid artery stenosis” or “CAAD” or “transient ischemic attack” or “TIA” or “peripheral arterial disease” or “PAD” or “peripheral artery occlusive disease” or “PAOD” or “renal artery stenosis” or “RAS” or “retinal artery occlusion” or “RAO” or “aortic aneurysm” or “atherosclerosis”. In addition, all references cited were reviewed to identify additional studies. If two or more studies sharing the same studied populations, the one with the small size was abandoned. Two reviewers (JW and QZL) searched the above databases independently. Decisions were compared and disagreements about study selection were resolved by involving a third reviewer (RXY). The search was limited to English and Chinese language papers. There was no restriction on time period, sample size, or population.

### 2.2. Inclusion and Exclusion Criteria

To be included in the present meta-analysis, the studies had to comply with the following major criteria: (1) case-control or cohort studies evaluating the associations between the* CX3CR1* T280M and V249I polymorphisms and AS risk; (2) published studies with full text articles; (3) sufficient published data for calculating odds ratios (ORs) with their 95% confidence intervals (CIs); (4) study population being adults; (5) the diagnosis of ischemic heart disease (CAD and AMI) being accorded with the result of coronary angiography, criteria of World Health Organization (WHO), criteria of European Society of Cardiology (ESC), or criteria of American College of Cardiology/American Heart Association (ACC/AHA); the diagnosis of ICVD is accorded with result of computed tomography (CT) or magnetic resonance imaging (MRI); the diagnosis of carotid atherosclerosis (CAA) was assessed by ultrasound color Doppler (USCD), and peripheral arterial disease (PAD) was diagnosed by the following criteria: clinical symptoms of PAD (intermittent claudication, rest pain, or gangrene) accompanied by an ankle-brachial-index and significant stenosis of leg arteries confirmed by FCDS and/or angiography.

Studies were excluded if they were (1) review or meta-analysis; (2) not conducted in humans; (3) duplicate studies.

### 2.3. Data Extraction

Data, including name of the first author, year of publication, study population (country, ethnicity), study type (case-control and cohort study), type of atherosclerotic disease, source of controls (population-based studies and hospital-based studies), sample size (total numbers of cases and controls), and number of genotypes in cases and controls, were extracted from each study by two reviewers independently (JW and QZL) according to the prespecified inclusion criteria. Studies that reported a comparison of two case samples, such as different age groups, were included in the meta-analysis as independent studies. Decisions were compared and disagreements about study selection were resolved by consensus or by involving a third reviewer (RXY).

### 2.4. Quality Assessment for Individual Studies

The quality of the individual studies was evaluated and scored by two reviewers independently based on the Newcastle-Ottawa Scale (NOS) [46]. Each study was assessed based on three broad perspectives: selection, comparability, and exposure, and each satisfactory answer received one point. The NOS ranges between zero (none of the quality criterion was met) up to nine stars (all the quality criteria were met), and the high-quality study was considered as the one with a score higher than six. The reviewer (RXY) examined the results, and a consensus was reached.

### 2.5. GRADE Quality Assessment

GRADE (grades of recommendation, assessment, development and evaluation) approach was adopted to grade quality of evidence for each association [47]. The GRADE system included level of evidence: (1) high quality, we are very confident that the true effect lies close to that of the estimate of the effect; (2) moderate quality, we are moderately confident in the effect estimate: the true effect is likely to be close to the estimate of the effect, but there is a possibility that it is substantially different; (3) low quality, our confidence in the effect estimate is limited: the true effect may be substantially different from the estimate of the effect; and (4) very low quality, we have very little confidence in the effect estimate: the true effect is likely to be substantially different from the estimate of effect. Two reviewers (JW and QZL) assessed quality independently and solved disagreement by discussion.

### 2.6. Statistical Analysis

For the controls of each study, Hardy-Weinberg equilibrium (HWE) was assessed using the chi-square test (*P* < 0.05 was considered significant deviation from HWE). We performed a haplotype analysis based on the genotype data, the haplotype frequencies were calculated by CubeX analysis software for each study separately and for the whole sample [48]. The strength of associations between the* CX3CR1* T280M and V249I polymorphisms and AS risk was assessed by ORs with 95% CIs. The pooled ORs were performed for dominant model (T/M + M/M* versus *T/T for T280M; V/I + I/I* versus *V/V for V249I), recessive model (M/M* versus *T/T + T/M for T280M; I/I* versus *V/V + V/I for V249I), codominant model (T/M* versus *T/T + M/M for T280M; V/I* versus *V/V + I/I for V249I), additive model (M/M* versus *T/T for T280M; I/I* versus *V/V for V249I), and allelic model (M allele* versus* T allele for T280M; I allele* versus* V allele for V249I). Bonferroni correction was used to control for the multiple testing in view of five genetic models under investigation (significance was set at 0.05/5 = 0.01), then another statistical significance was set as *P* < 0.05.

Heterogeneity across individual studies was calculated using Cochran's *Q* statistic (*P*_*Q*_) and the *I* ^2^ statistic. Values for *P*_*Q*_ < 0.10 and *I* ^2^ > 50% indicate a presence of heterogeneity among studies [49, 50], and the random-effects model was used for the meta-analysis. Otherwise, the fixed-effects model was used. Subgroup analysis was used to alleviate the heterogeneity based on ethnicity (Asian and Caucasians), the type of disease (CAD, ICVD, CAA, and PAD), and source of controls (population-based and hospital-based).

Sensitivity analyses were performed based on HWE (studies without HWE were excluded) and NOS score (studies with score ≤ 6 were excluded). Begg's funnel plot and Egger's regression test were conducted to identify possible publication bias in the current meta-analysis (*P* < 0.05 was considered representative of statistically significant publication bias). The analyses were performed using Stata software11.0 (StataCorp LP, College Station, USA).

## 3. Results

### 3.1. Study Characteristics

**Figure 1:**
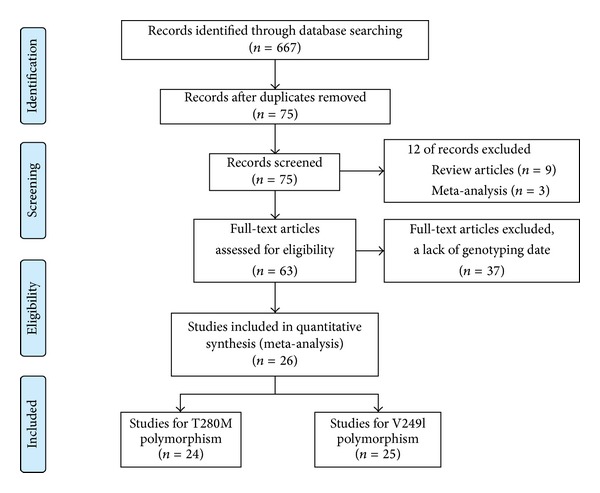
Flow chart showing study selection process.

The present study met the PRISMA statements and PRISMA flow chart (Checklist S1 available online at http://dx.doi.org/10.1155/2014/913678 and Figure 1). A total of 667 articles were identified after searching. After careful review, 26 articles involving 49 studies (24 studies for T280M and 25 studies for V249I polymorphisms) met the inclusion criteria and were selected in this meta-analysis [6–31]; one duplicate study was cut [18]. For the* CX3CR1* T280M polymorphism, 7732 AS cases and 5905 controls were included to assess the association between the variant and AS risk [6–12, 15–19, 21–28, 30, 31]. For the* CX3CR1* V249I polymorphism, 7952 AS cases and 6035 controls were included to assess the association between the variant and AS risk [6–12, 15–24, 26–31]. Main characteristics of the included studies were listed in Tables 1 and 2. The most commonly atherosclerotic disease included in the present meta-analysis was CAD in 21 studies. In addition, there were 8 studies involving carotid atherosclerosis, 8 studies involving cerebral infarction, 6 studies involving AMI, 2 studies involving ICVD, 2 studies involving ischemic stroke (IS), and 2 studies involving PAD. There were 24 studies of Asians and 25 studies of Caucasians. Three studies did not follow the HWE [8, 23, 26]. The results of GRADE were shown in Table S1.

### 3.2. Genotype-Phenotype Association

**Figure 2:**
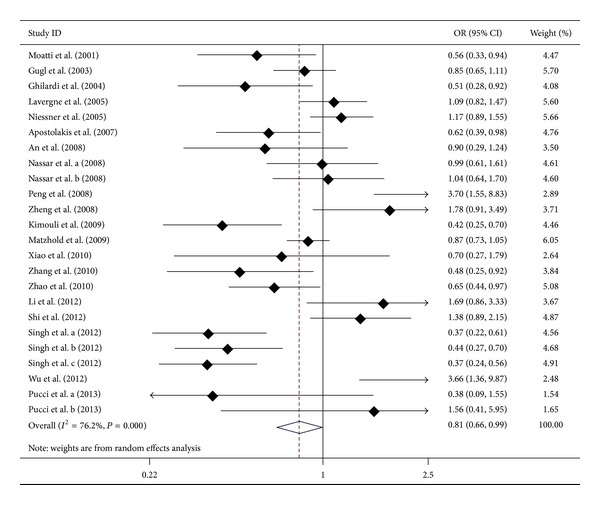
Forest plot for CX3CR1 T280M polymorphism and AS risk (TM* versus* TT).

As shown in Table 2, the overall results showed no significant association between the* CX3CR1* T280M polymorphism and the susceptibility to AS in five genetic models (*P* > 0.01 for all). When we performed a subgroup analysis, there was significant association between T280M polymorphism and the susceptibility to AS in the CAD group in dominant model (*P* < 0.01), codominant model (*P* < 0.01), additive model (*P* < 0.01), and allelic model (*P* < 0.01), but not in recessive model (*P* > 0.01), suggesting that the 280M allele carriers decreased the risk of CAD in the heterozygous state. The significant association between T280M polymorphism and the susceptibility to AS was also found in the ICVD group in recessive model (*P* < 0.01) and additive model (*P* < 0.01), but not in dominant model (*P* > 0.01), codominant model (*P* > 0.01), or allelic model (*P* > 0.01), suggesting that the 280M allele carriers increased the risk of ICVD in homozygote state. Then, we considered the TT genotype as the baseline risk, we found TM genotype was a protective role for AS (OR = 0.81, 95% CI = 0.66–0.99, *P* = 0.04; Table 3 and Figure 2), subgroup analysis showed that the TM genotype was a protective role for CAD (OR = 0.67, 95% CI = 0.52–0.87, *P* < 0.01; Table 3 and Figure S1), and MM genotype was a risk factor for ICVD (OR = 2.88, 95% CI = 1.64–5.04, *P* < 0.001; Table 3 and Figure S2). There was no association between the T280M polymorphism and the susceptibility to AS in the other groups (Tables 2 and 3).

**Figure 3:**
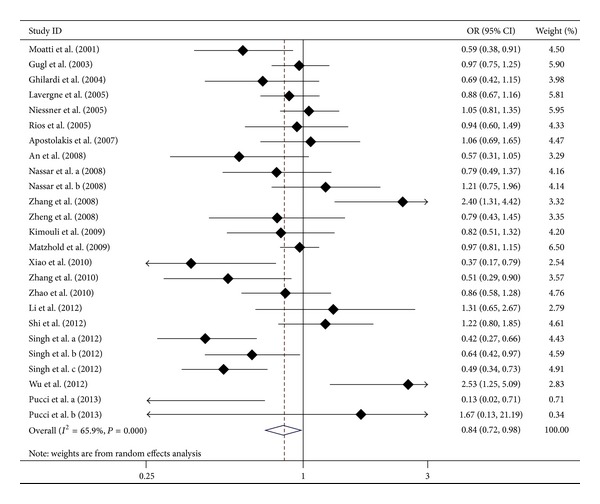
Forest plot for CX3CR1 V249I polymorphism and AS risk (VI* versus* VV).

The overall results showed no significant association between the* CX3CR1* V249I polymorphism and the susceptibility to AS in five genetic models (*P* > 0.01 for all, Table 2). Subgroup analysis showed significant association between V249I polymorphism and the susceptibility to AS in CAD group in dominant model (*P* < 0.01), codominant model (*P* < 0.01), additive model (*P* < 0.01), and allelic model (*P* < 0.01), but not in recessive model (*P* > 0.01), suggesting that the 249I alleles decreased the risk of CAD in heterozygote state. Significant associations were also found between this variant and the susceptibility to AS in the population-based (PB) group in recessive and additive models (*P* < 0.01 for each). Then, we considered the VV genotype as the baseline risk; we found VI genotype was a protective role for AS (OR = 0.84, 95% CI = 0.72–0.98, *P* = 0.02; Table 3 and Figure 3); subgroup analysis showed that the VI genotype was a protective role for CAD (OR = 0.72, 95% CI = 0.59–0.90, *P* < 0.01; Table 3 and Figure S3). There was no association between V249I polymorphism and the susceptibility to AS in the other groups (Tables 2 and 3).

For V249I-T280M combined genotype (Table S2), when we took VVTT genotype as a baseline risk, significant discrepancies were found in VVTM genotype (OR = 11.95, 95% CI = 5.00–28.58, *P* < 0.001; Table 3), suggesting that the VVTM genotype was a risk factor for AS, but VVTM was a very rare genotype and may not have potential clinical significance. Significant association was also found in the VITM (OR = 0.64, 95% CI = 0.50–0.82, *P* < 0.001) and IITM genotypes (OR = 0.69, 95% CI = 0.52–0.90, *P* < 0.01), suggesting that the VITM and IITM genotypes were protective roles for AS (Table 3).

### 3.3. Linkage Disequilibrium Analysis

There were nine combined genotypes in Table S2; six studies identified the extremely rare VVTM genotype. Linkage disequilibrium (LD) analysis showed a strong association between the T280M and V249I polymorphisms (Table 4). Thirteen of the fourteen studies conclusively found complete LD; an association analysis of the whole sample (cases and controls) indicated strong (but not complete) LD between the two loci (*D*′ = 0.9950,  *r* ^2^ = 0.5394).

### 3.4. Haplotype-Phenotype Association

Haplotype frequencies of each study and the total sample are reported in Table 4, the strong LD between the V249I and T280M polymorphisms resulted in three predominant haplotypes and the extremely rare 249V-280M; the latter haplotype accounts for less than 0.5% of the entire population's gene pool. We assessed the effect of the 249V-280M, 249I-280T, and 249I-280M haplotypes on the risk for AS independently taking the 249V-280T haplotype as a baseline risk. A significant predominance of the 249I-280M haplotype was observed in the control population compared to case subjects (OR = 0.63, 95% CI: 0.50–0.81, *P* < 0.001; Table 3). No association was observed between the 249I-280T haplotype and susceptibility to AS (OR = 1.09, 95% CI: 0.97–1.21, *P* = 0.14; Table 3).

### 3.5. Sensitivity Analysis

Sensitivity analyses were conducted to determine whether modification of the inclusion criteria of the meta-analysis affected the final results. The included studies were limited to those conforming to HWE or the high quality studies (NOS score ≥ 7); the corresponding pooled ORs were not materially altered, either for the* CX3CR1* T280M polymorphism or for the V249I polymorphism. This suggested that the overall results of this meta-analysis were statistically robust. The main results of sensitivity analyses are shown in Table 2.

### 3.6. Heterogeneity Analysis

For both* CX3CR1 *T280M and V249I polymorphisms, significant heterogeneity existed in the overall comparisons in five genetic models. We performed subgroup analyses based on the ethnic (Caucasians and Asian), atherosclerotic diseases (CAD, PAD, CAA, and ICVD), and source of control (hospital-based or population-based); heterogeneity was distinctly reduced; although it was still significant, we could not point out other possible sources of heterogeneity. The data were shown in Table S3.

For the V249I-T280M combined polymorphism, when the VVTT genotype was taken as the baseline risk, we found significant heterogeneity in the VITM genotype (*P*_*Q*_ < 0.1, *I* ^2^ = 69.2%). When the 249V-280T haplotype was taken as the baseline risk, we showed obvious heterogeneity in the 249I-280 M haplotype (*P*_*Q*_ < 0.1, *I* ^2^ = 82.2%; Table 3).

### 3.7. Publication Bias

**Figure 4:**
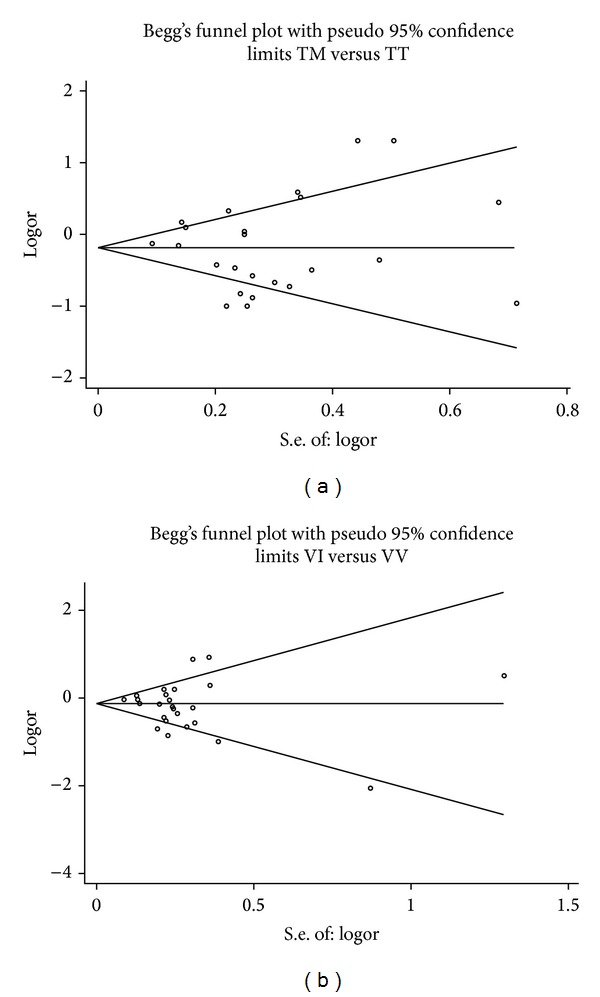
Begg's funnel plots for CX3CR1 T280M and V249I polymorphisms and AS risk. (a) TM* versus* TT for T280M polymorphism; (b) VI* versus* VV for V249I polymorphism.

Begg's funnel plot and Egger's regression test were performed to assess potential publication bias. For the* CX3CR1* T280M polymorphism, visual inspection of the funnel plot (Figure 4(a)) displays symmetrical distribution of OR estimations, suggesting no publication bias. In addition, the results of Egger's regression test also provided evidence for no publication bias (TM* versus* TT, *P* > 0.05 for all genetic models). For the* CX3CR1* V249I polymorphism, no obvious asymmetry was observed in any genetic model according to the visual assessment of funnel plot (Figure 4(b)). The results of Egger's regression test did not provide any statistical evidence for publication bias (VI* versus* VV, *P* > 0.05 for all genetic models).

## 4. Discussion

In this study, twenty-five articles involving the associations between the* CX3CR1 *polymorphisms and AS risk were included in the final meta-analysis. According to the GRADE approach, the quality of the evidence of* CX3CR1* T280M polymorphism was very low in dominant model and allelic model, moderate in recessive model and additive model, and low in codominant model (Table S1). For the* CX3CR1* V249I polymorphism, GRADE suggested that the quality of the evidence was moderate in recessive model, codominant model, and additive model, low in dominant model, and very low in allelic model (Table S1).

The overall findings showed that there was no association between the* CX3CR1* polymorphisms and the risk of AS in five genetic models. To make a more comprehensive analysis, subgroup analyses were performed based on ethnicity, atherosclerotic disease, and source of controls. For the* CX3CR1* T280M polymorphism, Apostolakis et al. [43] showed that the 280M allele carriers reduced the risk of CAD in heterozygote state. In the present study, we also found atheroprotective effect to AS in the CAD group. In addition, we found that the 280M allele carriers increased the risk of ICVD in homozygote state. For the* CX3CR1* V249I polymorphism, significant associations were found between this variant and the atheroprotective effect on AS in CAD and PB groups. These results suggested that the 249I allele carriers reduced the risk of CAD in heterozygote state. The above statistical results were based on Bonferroni correction to control for the multiple testing in view of under investigation.

For the combined genotype, the VITM and IITM genotypes played an atheroprotective effect on AS. The combined VVTM genotype was more common in the cases than in the controls; however, the rarity of the VVTM genotype makes any conclusion rather unsafe. LD analysis indicated a strong association between T280M and V249I, and a protective role of the 249I-280M haplotype was also observed in the control population compared to case subjects. No association was observed between the 249I-280T haplotype and the susceptibility to AS. These results were consistent with those of a previous study [43].

Considering the studies without HWE or with low NOS score may influence the overall results, subsequent sensitivity analyses restricted to the studies with HWE or high NOS score were performed, but no corresponding pooled OR was materially altered in the dominant, recessive, codominant, additive, and allelic models. These results suggested that the studies without HWE or low score should not be considered as a factor influencing the overall results.

Heterogeneity of the included studies is the most important drawback when the genotypic data were analyzed, either for the* CX3CR1* T280M or for the* CX3CR1* V249I polymorphism. Heterogeneity should not be ignored and should be carefully factored in the interpretation of the final results. For the* CX3CR1* T280M and V249I polymorphisms, the heterogeneity can partly be explained by the ethnicity (Caucasians and Asian), subtype of atherosclerotic diseases (CAD, CAA, and ICVD), and source of control (hospital-based and population-based).

However, some limitations of this meta-analysis should be acknowledged. Firstly, there was significant heterogeneity in this meta-analysis. Heterogeneity may affect the precision of overall results, despite the use of appropriate meta-analytic techniques with random-effects model. Secondly, in the subgroup analyses, the sample sizes in some subgroup, such as the PAD and CAA groups of the* CX3CR1* T280M and V249I polymorphisms, were relatively small, not having enough statistical power to explore the real association. Thirdly, AS is a complex disease and involves potential interactions of gene-environment. However, many eligible studies included in this meta-analysis did not consider the environmental factors. Therefore, studies with larger sample sizes and better design are needed.

## 5. Conclusion

The present meta-analysis suggested that the* CX3CR1* 280M and 249I allele carriers had atheroprotective roles on AS in heterozygote state, and the 280M allele carriers were associated with the susceptibility to AS in homozygote state. The combined genotypes of VITM and IITM also had atheroprotective roles on AS. Consequently, this effect may be attributed to the haplotype of 249I-280M. However, the results should be interpreted with caution because of its limitations. Further studies with large sample size, especially with the consideration of gene-gene and gene-environment interactions, are needed to confirm our findings.

## Supplementary Material


Checklist S1: The PRISMA 2009 checklist. Figure S1: Forest plot for CX3CR1 T280M polymorphism and AS risk in CAD group (TM versus TT). Figure S2: Forest plot for CX3CR1 T280M polymorphism and AS risk in ICVD group (MM versus TT). Figure S3: Forest plot for CX3CR1 V249I polymorphism and AS risk in CAD group (VI versus VV). Table S1: GRADE profile evidence of the included studies. Table S2: Genotype distribution in cases and controls. Table S3: Heterogeneity test of CX3CR1 T280M and V249I polymorphisms and risk of AS in each subgroup.

## Figures and Tables

**Table 1:** Characteristics of studies included in this meta-analysis.

Position	First author	Year	Country	Study type	Disease	Source of control	SS (Case/Control)	Genotypes distribution (case/control)					HWE Y/N (*P*)	Score
								T/T	T/M	M/M	T	M		
T280M	Moatti [15]	2001	France	CCS	CAD	HB	151/249	123/179	25/65	3/5	271/423	31/75	Y (0.748)	8
	Gugl [8]	2003	Austria	CCS	PAD	HB	492/503	333/326	143/165	16/12	809/817	175/189	Y (0.093)	8
	Ghilardi [7]	2004	Italia	CCS	CAA	HB	108/204	87/142	18/58	3/4	192/342	24/66	Y (0.490)	8
	Lavergne [10]	2005	France	CCS	CI	HB	460/469	314/338	125/123	21/8	753/799	167/139	Y (0.400)	7
	Niessner [17, 18]	2005	Austria	CCS	CAD	HB	720/432	514/319	189/100	17/13	1217/738	223/126	Y (0.141)	7
	Apostolakis [43]	2007	Greece	CCS	CAD	HB	210/165	159/109	48/53	3/3	366/271	54/59	Y (0.228)	7
	An [24]	2008	China	CCS	CAD	HB	108/80	88/59	18/20	2/1	194/138	22/22	Y (0.629)	6
	Nassar-a [16]	2008	Canada	CCS	CAD	PB	149/149	97/94	49/48	3/7	243/236	55/62	Y (0.784)	8
	Nassar-b [16]	2008	Canada	CCS	CAD	PB	150/149	92/94	49/48	9/7	233/236	67/62	Y (0.784)	8
	Peng [25]	2008	China	CCS	ICVD	PB	165/150	138/143	25/7	2/0	301/293	29/7	Y (0.770)	8
	Zheng [31]	2008	China	CCS	CAA	HB	117/93	77/73	32/17	8/3	186/163	48/23	Y (0.131)	6
	Kimouli [9]	2009	Greece	CCS	CAA	PB	150/151	112/87	31/58	7/6	255/232	45/70	Y (0.334)	8
	Matzhold [12]	2009	Austria	CCS	CAD	PB	2565/728	1797/483	706/217	59/26	4300/1183	824/269	Y (0.790)	7
	Xiao [28]	2010	China	CCS	CAD	PB	139/90	129/81	10/9	0/0	268/171	10/9	Y (0.618)	7
	Zhang [30]	2010	China	CCS	CI	PB	120/102	98/71	20/30	2/1	216/172	24/32	Y (0.258)	7
	Zhao [23]	2010	China	CCS	CAA	HB	318/292	246/201	56/70	16/21	548/472	88/112	N (0.000)	7
	Li [11]	2012	China	CCS	CI	PB	308/294	284/280	24/14	0/0	592/574	24/14	Y (0.676)	8
	Shi [26]	2012	China	CCS	CI	HB	563/563	493/518	50/38	20/7	1036/1074	90/52	N (0.000)	7
	Singh-a [21, 22]	2012	India	CCS	CAD	PB	152/300	126/191	24/99	2/10	276/481	28/119	Y (0.513)	8
	Singh-b [21, 22]	2012	India	CCS	CAD	PB	156/300	124/191	28/99	4/10	276/481	36/119	Y (0.513)	8
	Singh-c [21, 22]	2012	India	CCS	AMI	PB	230/300	190/191	36/99	4/10	416/481	44/119	Y (0.513)	7
	Wu [27]	2012	China	CCS	IS	PB	163/100	135/95	26/5	2/0	296/195	30/5	Y (0.798)	7
	Pucci-a [19]	2013	Italy	CCS	AMI	HB	25/22	21/14	4/7	0/1	46/35	4/9	Y (0.917)	7
	Pucci-b [19]	2013	Italy	CCS	AMI	HB	16/22	9/14	7/7	0/1	25/35	7/9	Y (0.917)	7

Position	First author	Year	Country	Study type	Disease	Source of control	SS (case/control)	Genotypes distribution (case/control)					HWE Y/N (*P*)	Score
								V/V	V/I	I/I	V	I		

V249I	Moatti [15]	2001	France	CCS	CAD	HB	151/249	97/126	47/104	7/19	241/356	61/142	Y (0.699)	8
	Gugl [8]	2003	Austria	CCS	PAD	HB	492/503	261/268	198/210	33/25	720/746	264/260	N (0.045)	8
	Ghilardi [7]	2004	Italia	CCS	CAA	HB	108/204	63/108	34/84	11/12	160/300	56/108	Y (0.409)	8
	Lavergne [10]	2005	France	CCS	CI	HB	458/468	234/236	178/203	46/29	646/675	270/261	Y (0.089)	7
	Niessner [17, 18]	2005	Austria	CCS	CAD	HB	720/432	395/246	271/161	54/25	1061/653	379/211	Y (0.842)	7
	Rios [20]	2005	Brazil	CS	CAD	HB	219/129	120/72	80/51	19/6	320/195	118/63	Y (0.420)	7
	Apostolakis [43]	2007	Greece	CCS	CAD	HB	210/165	111/86	81/59	18/20	303/231	117/99	Y (0.056)	7
	An [24]	2008	China	CCS	CAD	HB	108/80	70/41	33/34	5/5	173/116	43/44	Y (0.556)	6
	Nassar-a [16]	2008	Canada	CCS	CAD	PB	149/149	73/63	63/69	13/17	209/195	89/103	Y (0.772)	7
	Nassar-b [16]	2008	Canada	CCS	CAD	PB	150/149	58/63	77/69	15/17	193/195	107/103	Y (0.772)	8
	Zhang [29]	2008	China	CCS	ICVD	PB	165/150	122/132	40/18	3/0	284/282	46/18	Y (0.434)	8
	Zheng [31]	2008	China	CCS	CAA	HB	117/93	69/53	33/32	15/8	171/138	63/48	Y (0.328)	6
	Kimouli [9]	2009	Greece	CCS	CAA	PB	150/151	82/73	59/64	9/14	223/210	77/92	Y (0.996)	8
	Matzhold [12]	2009	Austria	CCS	CAD	PB	2565/728	1374/372	1012/283	179/73	3760/1027	1370/429	Y (0.081)	7
	Xiao [28]	2010	China	CCS	CAD	PB	139/90	126/68	13/19	0/3	265/155	13/25	Y (0.265)	7
	Zhang [30]	2010	China	CCS	CI	PB	120/102	81/51	35/43	4/8	197/145	43/59	Y (0.798)	7
	Zhao [23]	2010	China	CCS	CAA	HB	318/292	194/164	66/65	58/63	454/393	182/191	N (0.000)	7
	Li [11]	2012	China	CCS	CI	PB	308/294	289/280	19/14	0/0	597/574	19/14	Y (0.676)	8
	Shi [26]	2012	China	CCS	CI	HB	563/563	497/512	52/44	14/7	1046/1068	80/58	N (0.000)	7
	Singh-a [21, 22]	2012	India	CCS	CAD	PB	152/300	111/150	37/118	4/32	259/418	45/182	Y (0.230)	8
	Singh-b [21, 22]	2012	India	CCS	CAD	PB	156/300	96/150	48/118	12/32	240/418	72/182	Y (0.230)	8
	Singh-c [21, 22]	2012	India	CCS	AMI	PB	230/300	157/150	61/118	12/32	375/419	85/182	Y (0.230)	7
	Wu [27]	2012	China	CCS	IS	PB	163/100	119/88	41/12	3/0	279/188	47/12	Y (0.523)	7
	Pucci-a [19]	2013	Italy	CCS	AMI	HB	25/22	13/2	10/12	2/8	36/16	14/28	Y (0.402)	7
	Pucci-b [19]	2013	Italy	CCS	AMI	HB	16/22	1/2	10/12	5/8	12/16	20/28	Y (0.402)	7

CCS: case-control study, CS: cohort study, SS: sample size, PB: population-based, HB: hospital-based, HWE: Hardy-Weinberg equilibrium, Y: yes, N: no, AMI: acute myocardial infarction, CAA: carotid artery atherosclerosis, CAD: coronary artery disease, CI: cerebral infarction, ICVD: ischemic cerebrovascular disease, IS: ischemic stroke, PAD: peripheral arterial disease.

**Table 2:** Meta-analyses of *CX3CR1* T280M and V249I polymorphisms and risk of AS in each subgroup.

Position	SS (case/control)	Dominant model		Recessive model		Codominant model		Additive model		Allelic model	
		OR (95% CI)	*P*	OR (95% CI)	*P*	OR (95% CI)	*P*	OR (95% CI)	*P*	OR (95% CI)	*P*
Overall analysis											
T280M	7732/5905	0.83 [0.68, 1.02]	0.08	1.01 [0.82, 1.26]	0.91	0.81 [0.67, 0.99]	0.04	0.96 [0.77, 1.19]	0.72	0.88 [0.73, 1.05]	0.16
V249I	7952/6035	0.84 [0.71, 0.98]	0.03	0.88 [0.69, 1.11]	0.27	0.87 [0.76, 0.99]	0.04	0.81 [0.62, 1.05]	0.12	0.86 [0.75, 0.99]	0.04

Subgroup analysis based on ethnicity											
T280M (C)	5193/3241	0.83 [0.69, 0.99]	0.04	0.97 [0.74, 1.28]	0.85	0.82 [0.68, 0.98]	0.03	0.94 [0.71, 1.23]	0.64	0.87 [0.75, 1.01]	0.06
T280M (A)	2539/2664	0.92 [0.60, 1.43]	0.72	1.09 [0.76, 1.56]	0.66	0.88 [0.58, 1.32]	0.54	1.00 [0.70, 1.44]	0.99	0.98 [0.66, 1.47]	0.93
V249I (C)	5413/3371	0.91 [0.81, 1.03]	0.15	0.96 [0.72, 1.28]	0.79	0.94 [0.86, 1.03]	0.18	0.92 [0.67, 1.27]	0.62	0.92 [0.82, 1.04]	0.19
V249I (A)	2539/2664	0.80 [0.57, 1.14]	0.22	0.75 [0.48, 1.16]	0.19	0.84 [0.63, 1.13]	0.25	0.66 [0.40, 1.09]	0.10	0.84 [0.62, 1.15]	0.28

Subgroup analysis based on type of diseases											
T280M (CAD)	4768/2984	0.67 [0.52, 0.85]	<0.01	0.69 [0.51, 0.93]	0.01	0.68 [0.53, 0.88]	<0.01	0.64 [0.48, 0.87]	<0.01	0.70 [0.58, 0.86]	<0.01
T280M (PAD)	492/503	0.88 [0.68, 1.14]	0.34	1.38 [0.64, 2.94]	0.41	0.84 [0.64, 1.10]	0.20	1.31 [0.61, 2.80]	0.49	0.94 [0.75, 1.17]	0.56
T280M (CAA)	693/740	0.73 [0.43, 1.23]	0.23	0.98 [0.60, 1.60]	0.94	0.68 [0.41, 1.14]	0.15	0.89 [0.55, 1.46]	0.65	0.81 [0.52, 1.25]	0.33
T280M (ICVD)	1779/1678	1.58 [0.98, 2.53]	0.06	2.83 [1.62, 4.95]	<0.01	1.43 [0.88, 2.31]	0.15	2.90 [1.65, 5.07]	<0.01	1.65 [1.06, 2.55]	0.03
V249I (CAD)	4990/3115	0.69 [0.55, 0.86]	<0.01	0.70 [0.53, 0.92]	0.01	0.79 [0.66, 0.94]	<0.01	0.62 [0.43, 0.87]	<0.01	0.72 [0.59, 0.87]	<0.01
V249I (PAD)	492/503	1.01 [0.79, 1.30]	0.94	1.38 [0.81, 2.35]	0.24	0.94 [0.73, 1.21]	0.63	1.36 [0.78, 2.34]	0.28	1.05 [0.86, 1.28]	0.62
V249I (CAA)	693/740	0.82 [0.66, 1.01]	0.07	1.02 [0.65, 1.60]	0.94	0.82 [0.65, 1.03]	0.09	0.93 [0.61, 1.40]	0.71	0.87 [0.74, 1.03]	0.11
V249I (ICVD)	1777/1677	1.29 [0.83, 2.01]	0.25	1.50 [0.78, 2.90]	0.23	1.22 [0.80, 1.85]	0.35	1.44 [0.65, 3.17]	0.37	1.33 [0.89, 1.99]	0.16

Subgroup analysis based on source of controls											
T280M (HB)	3288/3094	0.89 [0.71, 1.13]	0.34	1.30 [0.97, 1.74]	0.08	0.86 [0.70, 1.06]	0.16	1.27 [0.95, 1.70]	0.11	0.94 [0.75, 1.17]	0.56
T280M (PB)	4444/2811	0.81 [0.57, 1.14]	0.22	0.74 [0.54, 1.03]	0.07	0.80 [0.57, 1.13]	0.21	0.68 [0.49, 0.94]	0.02	0.84 [0.63, 1.13]	0.25
V249I (HB)	3505/3222	0.91 [0.78, 1.06]	0.22	1.13 [0.86, 1.49]	0.37	0.89 [0.80, 1.00]	0.04	1.08 [0.79, 1.48]	0.64	0.94 [0.82, 1.09]	0.42
V249I (PB)	4447/2813	0.80 [0.59, 1.09]	0.15	0.62 [0.48, 0.81]	<0.01	0.88 [0.68, 1.15]	0.35	0.55 [0.38, 0.78]	<0.01	0.81 [0.63, 1.04]	0.10

Sensitivity analysis											
T280M (BS)	7507/5732	0.81 [0.66, 1.00]	0.05	0.99 [0.79, 1.23]	0.90	0.80 [0.65, 0.98]	0.03	0.93 [0.75, 1.16]	0.52	0.86 [0.71, 1.03]	0.10
T280M (BH)	6851/5050	0.82 [0.66, 1.00]	0.06	0.97 [0.74, 1.23]	0.79	0.80 [0.65, 0.99]	0.04	0.91 [0.72, 1.16]	0.47	0.85 [0.71, 1.03]	0.09
V249I (BS)	7727/5862	0.84 [0.71, 1.00]	0.05	0.86 [0.67, 1.10]	0.23	0.89 [0.77, 1.02]	0.09	0.79 [0.60, 1.05]	0.11	0.86 [0.74, 1.00]	0.06
V249I (BH)	6579/4677	0.80 [0.67, 0.97]	0.02	0.82 [0.62, 1.07]	0.14	0.85 [0.73, 0.99]	0.04	0.74 [0.54, 1.00]	0.05	0.83 [0.71, 0.98]	0.03

A: Asians; C: Caucasians; CAD: coronary artery disease; CAA: carotid artery atherosclerosis; ICVD: ischemic cerebrovascular disease; PAD: peripheral arterial disease; PB: population-based, HB: hospital-based; BS: based on score (studies with score ≤ 6 were excluded); BH: based on HWE (studies without HWE were excluded). Bonferroni correction was used to control for the multiple testing in view of five genetic models under investigation (significance was set at 0.05/5 = 0.01).

**Table 3:** The independent effect of each genotype/haplotype on susceptibility to atherosclerosis.

Genotype	Cases (*n*)	Controls (*n*)	Baseline risk	OR	95% CI	*P*	*I* ^2^ (%)	*P*_*Q*_
VVTT	1923	2601		1				
VVTM	49	3	VVTT	11.95	5.00–28.58	<0.001	0	0.44
VITT	516	700	VVTT	1.11	0.96–1.27	0.16	31.4	0.13
VITM	605	1211	VVTT	0.64	0.50–0.82	<0.001	69.2	<0.1
IITT	64	76	VVTT	1.20	0.83–1.74	0.33	0	0.46
IITM	100	182	VVTT	0.69	0.52–0.90	<0.01	41.7	<0.1
IIMM	87	151	VVTT	0.76	0.57–1.02	0.06	0	0.79
VV	4813	3556		1				
VI	2598	2016	VV	0.84	0.72–0.98	0.02	65.9	<0.1
VI (CAD)	1843	1227	VV (CAD)	0.72	0.59–0.90	<0.01	68.1	<0.1
II	541	463	VV	0.81	0.62–1.05	0.12	61.6	<0.1
TT	5786	4293		1				
TM	1743	1456	TT	0.81	0.66–0.99	0.04	76.2	<0.1
TM (CAD)	1193	871	TT (CAD)	0.67	0.52–0.87	<0.01	72.2	<0.1
MM	203	156	TT	0.96	0.77–1.19	0.72	31.9	0.08
MM (ICVD)	47	16	TT (ICVD)	2.88	1.64–5.04	<0.001	0	0.92
VT	5377	6975		1				
VM	77	5	VT	11.47	5.34–24.64	<0.001	43.0	0.12
IT	814	1007	VT	1.09	0.97–1.21	0.14	0	0.55
IM	892	1665	VT	0.63	0.50–0.81	<0.001	82.2	<0.1

*P*_*Q*_, *P* value for Cochran's *Q* statistic.

**Table 4:** Estimated haplotype frequencies and linkage disequilibrium analysis of the included studies.

First author	Estimated haplotype frequencies (cases)				Estimated haplotype frequencies (controls)				*D*′^a^	*r* ^2^ ^a^
	V249 T280	V249 M280	I249 T280	I249 M280	V249 T280	V249 M280	I249 T280	I249 M280		
McDermott [13, 14]	0.7817	0	0.0838	0.1345	0.7042	0	0.0986	0.1972	1	0.5848
Moatti [15]	0.7980	0	0.0993	0.1026	0.7149	0	0.1345	0.1506	1	0.4445
Gugl [8]	0.7317	0	0.0904	0.1778	0.7416	0	0.0706	0.1879	1	0.6638
McDermott [13, 14]	0.7304	0	0.1324	0.1373	0.7018	0	0.1157	0.1825	1	0.5253
Ghilardi [7]	0.7407	0	0.1481	0.1111	0.7255	0	0.1225	0.1520	1	0.4736
Hattori [44]	0.9318	0.0256	0.0065	0.0361	0.9444	0.0082	0.0033	0.0441	0.9270	0.7743
Niessner [17, 18]	0.7332	0.0016	0.1113	0.1540	0.7564	0	0.0998	0.1439	1	0.5217
Apostolakis [6, 43]	0.7214	0	0.1500	0.1286	0.7000	0	0.1212	0.1788	1	0.508
An [24]	0.8241	0	0.0833	0.0926	0.7187	0	0.1250	0.1562	1	0.4733
Nassar-a [16]	0.7067	0.0171	0.1440	0.1321	0.6544	0	0.1376	0.2081	1	0.4974
Nassar-b [16]	0.6250	0.0203	0.1622	0.1925	0.6544	0	0.1376	0.2081	1	0.4974
Zhao [23]	0.7138	0	0.1478	0.1384	0.6729	0	0.1353	0.1918	1	0.4882
Singh-a, b [21, 22]	0.7559	0.0542	0.1402	0.0497	0.6967	0	0.1050	0.1983	1	0.5682
Singh-c [21, 22]	0.7739	0.0413	0.1304	0.0543	0.6967	0	0.1050	0.1983	1	0.5682

Total	0.7477	0.0091	0.1120	0.1312	0.7224	0.0006	0.1050	0.1720	0.9950	0.5394

^a^ *D*′ and *r* ^2^ statistics refer to control groups.
